# WHF Roadmap on Single Pill Combination Therapies

**DOI:** 10.5334/gh.1457

**Published:** 2025-08-29

**Authors:** Enrico G. Ferro, Gautam Satheesh, José Castellano, Albertino Damasceno, Okeoma Erojikwe, Mark Huffman, Vilma Irazola, Philip Joseph, Fernando Lanas, Elijah Ogola, Pedro Ordunez, Pablo Perel, Daniel Pineiro, Izabela Uchmanowicz, Orly Vardeny, Ruth Webster, Habib Gamra, Thomas Gaziano, Adrianna Murphy

**Affiliations:** 1 Richard A. and Susan F. Smith Center for Outcomes Research, Division of Cardiology, Beth Israel Deaconess Medical Center, Harvard Medical School, Boston, Massachusetts, United States; 2 Division of Cardiovascular Medicine, Beth Israel Deaconess Medical Center, Harvard, United States; 3 The George Institute for Global Health, Hyderabad, India; 4 The George Institute for Global Health, Sydney, Australia; 5 The University of Sydney, Sydney, New South Wales, Australia; 6 Spanish National Centre for Cardiovascular Research, Spain; 7 Eduardo Mondlane University, Mozambique; 8 Resolve to Save Lives, United States; 9 Washington University in St. Louis, St Louis, Missouri, United States; 10 Institute for Clinical Effectiveness and Health Policy (IECS), Argentina; 11 Population Health Research Institute, Hamilton Health Sciences and McMaster University, Hamilton, Ontario, Canada; 12 Universidad de La Frontera, Temuco, Chile; 13 University of Nairobi, Kenya; 14 Pan American Health Organization, United States; 15 London School of Hygiene and Tropical Medicine, London, United Kingdom; 16 World Heart Federation, Geneva, Switzerland; 17 Universidad de Buenos Aires, Argentina; 18 Wroclaw Medical University, Poland; 19 Minneapolis VA Center for Care Delivery and Outcomes Research, University of Minnesota, Minneapolis, MN, United States; 20 The George Institute for Global Health, Newtown, NSW, Australia; 21 Les Oliviers Medical Center, Sousse, Tunisia; 22 Harvard Medical School, Brigham & Women’s Hospital, Harvard T.H. Chan School of Public Health, United States; 23 Department of Health Service Research and Policy, Faculty of Public Health and Policy, London School of Hygiene and Tropical Medicine, London, United Kingdom

**Keywords:** Cardiovascular disease, single pill combinations, prevention, polypill

## Abstract

Cardiovascular diseases (CVDs) are the leading global cause of mortality, with treatment adherence posing a major barrier to effective prevention and control. Single pill combinations (SPCs), also known as fixed-dose combinations, simplify treatment by combining multiple agents into one pill, improving adherence and reducing cardiovascular risk. This World Heart Federation Roadmap synthesizes the latest clinical evidence and identifies key barriers to SPC implementation, including limited manufacturing, affordability, regulatory complexity, and inconsistent guideline inclusion. Drawing on global expert input and health systems analysis, the Roadmap outlines practical, context-specific solutions to improve access, scale-up, and integration of SPCs into national strategies, especially in low- and middle-income countries. It serves as a tool for policymakers, clinicians, and advocates to drive progress in aligning cardiovascular prevention efforts with evidence-based, people-centred care.

## Introduction

Cardiovascular diseases (CVDs) are the leading cause of death globally. In 2021, 20.5 million people died from a cardiovascular condition (1). Several causal pathways are associated with CVDs, including raised blood pressure, raised cholesterol levels, and platelet activation. Treatment with drugs from multiple therapeutic classes, therefore, is recommended to reduce morbidity and mortality from CVD.

While a combination of multiple therapeutics prevents morbidity and mortality from CVD, the resulting pill burden for patients can hamper adherence to treatment and health outcomes. For this reason, in 2001 the World Health Organization (WHO) built on the original idea proposed by Wald & Law in 2000 to assemble fixed-dose combinations (FDCs) to treat CVDs, i.e. to combine more than one active pharmaceutical ingredient into a single pill to target *one* specific condition (2). Over the last two decades, FDCs have been successfully implemented for certain cardiovascular conditions. These include high cholesterol levels (where combination therapy achieved better cholesterol control than monotherapy) (3,4) and high blood pressure, for which FDCs were included in the WHO Model List of Essential Medicines (EML) in 2019 to support adoption in national essential medicine lists around the world (5,6). FDCS are also used in treating several non-cardiovascular conditions, such as HIV, tuberculosis and malaria.

The term single pill combination (SPC) is now used as an alternative to FDC, as the concept of “fixed dose” does not capture that the SPC platform is designed to easily change the dose of each pharmaceutical ingredient in a pill (meaning they are not “fixed”). Further, the term “polypill” is sometimes utilized to indicate a specific type of FDC in which multiple drugs are combined in one single pill to simultaneously treat *more than one* condition (2).

Despite their differences, FDC, SPC and polypill are often used interchangeably, due to their similar technological features and shared potential to improve cardiovascular outcomes on a global scale. For the sake of simplicity, we use the term SPC throughout this document.

While SPCs have successfully helped treat conditions like high blood pressure, they remain underutilized for primary and secondary prevention of CVD – the focus of this Roadmap. Primary prevention refers to the treatment of people considered at sufficiently high risk of developing clinically significant atherosclerotic CVD (ASCVD), such as people with asymptomatic coronary atherosclerosis or elevated ASCVD risk factor levels. Secondary prevention refers to preventing a recurrent event among people who already have established ASCVD or have experienced a major adverse cardiovascular event (MACE), such as myocardial infarction or stroke.

**Figure 1:**
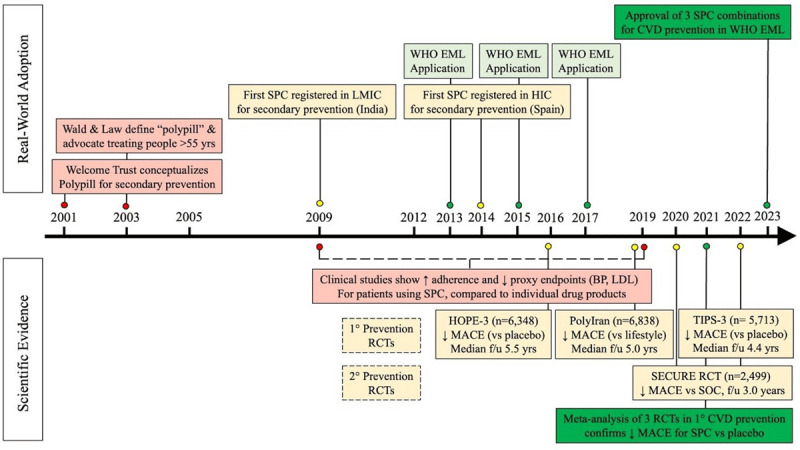
Development and Implementation of Single Pill Combinations for Cardiovascular Disease. **Figure Legend**. Milestones in the history of SPC for cardiovascular disease are listed chronologically and separated into two categories: scientific evidence to support the use of SPC in primary and secondary CVD prevention (bottom), and key events towards real-world adoption of SPC (top). The colour coding of the boxes is used to represent the progression in each of the two domains: from trials limited to intermediate outcomes (red) to trials evaluating clinical outcomes (yellow) to systematic meta-analyses (green) for the scientific evidence domain; and from SPC idea conceptualization (red) to manufacturing efforts (yellow) to policy implementation (green) for the real-world adoption domain. ***Acronyms:*** Blood pressure (BP); cardiovascular disease (CVD); essential medicine list (EML); single pill combination (SPC); high-income countries (HIC); low- and middle-income countries (LMIC); low-density lipoprotein (LDL); major adverse cardiovascular events (MACE); randomized controlled trial (RCT); standard of care (SOC).

In the last 20 years, the evidence base for the established benefits of SPC use in primary and secondary prevention of CVDs has grown (Figure 1). For primary ASCVD prevention, high-quality data demonstrate SPC’s efficacy and safety (7,8,9). For secondary ASCVD prevention, randomized trials show net clinical benefits of SPCs, further supporting clinical adoption and implementation (10).

Based on this evidence and updated results from large randomized trials (11,12), the World Heart Federation (WHF) partnered with other cardiovascular health scientists and advocates to successfully petition for the inclusion of cardiovascular SPCs in the 2023 WHO EML. Their inclusion has the potential to lower the burden of CVD through influencing government procurement efforts, professional guideline recommendations, and clinician adoption. Collectively, these forces could increase the demand for SPCs to the level needed to motivate industry manufacturing and insurance coverage efforts.

To seize on the momentum from 2023, WHF has developed this Roadmap to promote and support the adoption and implementation of SPCs for primary and secondary prevention of CVD, based on the priorities, capabilities, and resources of healthcare systems.

This WHF Roadmap builds on others released since 2014 to support public health policy in preventing, detecting, and managing CVDs. It will specifically address three CVD pathways – blood pressure, cholesterol levels, and platelet activation – for which SPCs have the most supportive evidence in terms of safety and efficacy, increased adherence and cost-effectiveness for patients and health systems (11,13,14,15,16). Importantly, this Roadmap applies equally to low- and middle-income countries (LMICs), where over 75% of CVD-related deaths occur, and to high-income countries (HICs), where in some cases age-standardized rates of CVD have started to rise again. In the near future, the SPC therapy approach to prevention can be readily translated to benefit other CVD pathways with increasing global burden, such as diabetes and heart failure with reduced ejection fraction, for which evidence of the benefits of SPCs is emerging (17,18,19).

In short, this WHF Roadmap will:

1. Summarize the evidence on the efficacy, safety, and cost-effectiveness of SPCs to treat and prevent select CVD events
2. Outline contemporary facilitators and barriers to availability, affordability, and adoption of SPCs
3. Propose actionable solutions for healthcare systems, including lessons learned from the implementation of non-cardiovascular SPC therapies

This Roadmap is aimed at stakeholders who collaborate to bridge the gap between knowledge and implementation of SPCs for CVD – including governments, professional societies, pharmaceutical manufacturers, insurance companies, clinicians and, the community of people living with and/or at risk for CVD.

## Clinical Evidence for SPCs

Randomized controlled trials (RCTs) of SPCs for CVD have primarily focused on the treatment of hypertension, dyslipidaemia, and atherosclerosis through the combination of at least two blood pressure lowering agents and a statin (with or without aspirin) in a single pill formulation. SPCs have been studied in both primary and secondary CVD prevention, and study outcomes include intermediate outcomes (i.e. medication adherence and risk factors like blood pressure and cholesterol levels) and clinical outcomes (i.e. stroke, myocardial infarction, and cardiovascular disease mortality). Four smaller RCTs (UMPIRE, IMPACT, Kanyini GAP and FOCUS) evaluated the impact of SPC on major adverse cardiovascular events (MACE) – although these trials were primarily designed and powered to study the effect of SPCs on CVD risk factors (e.g. blood pressure or cholesterol levels) (20,21,22). While none of these smaller trials (500–2,000 people prescribed SPC for primary or secondary CVD prevention) found a significant reduction in MACE with SPC compared to usual care or placebo, they were underpowered to detect this outcome. Therefore, these secondary analyses should be considered exploratory.

### Major adverse cardiovascular events (MACE)

Three large RCTs (HOPE-3, PolyIran, and TIPS-3) have tested an SPC strategy in adequately large populations (each with >5,000 people at risk of developing CVDs) and for sufficiently long follow-up time (>4 years) to achieve the power needed to show the impact of SPC in reducing the incidence of MACE compared to usual care or placebo, with a focus on participants who were free of CVD at baseline (Table 1). The three trials achieved an important level of geographic representation by collectively enrolling patients from 11 HICs and 15 LMICs (7,8,9). Of note, the HOPE-3 trial tested an SPC strategy, but effectively administered the cholesterol- and blood-pressure-lowering agents as separate pills; nonetheless, it was included in the meta-analysis, as it is one of only three trials testing an SPC strategy that was powered for clinical outcomes (7).

**Table 1:** Effects of SPC versus Control in Primary and Secondary CVD Prevention Trials.


PRIMARY PREVENTION OF CVD*				

	CONTROL GROUP N (%)	SPC GROUP N (%)	HR (95%CI)	p-VALUE

**SPC Strategy versus Control (Composite)**				

Randomized	9,088	9,074		

CV death, MI, stroke, revascularization	445 (4.9)	276 (3.0)	0.62 (0.53–0.73)	<0.0001

CV Death	227 (2.5)	144 (1.6)	0.65 (0.52–0.81)	<0.0001

MI	139 (1.5)	70 (0.8)	0.52 (0.38–0.70)	<0.0001

Stroke	141 (1.6)	83 (0.9)	0.59 (0.45–0.78)	0.0002

Revascularization	70 (0.8)	39 (0.4)	0.54 (0.36–0.80)	0.002

SPC Strategy versus Control (Subgroup: SPC with Aspirin)**				

Randomized	4,489	4,462		

CV death, MI, stroke, revascularization	217 (4.8)	115 (2.6)	0.53 (0.41–0.67)	<0.0001

CV Death	114 (2.5)	58 (1.3)	0.51 (0.37–0.72)	<0.0001

MI	89 (2.0)	42 (0.9)	0.47 (0.32–0.69)	0.0001

Stroke	73 (1.6)	36 (0.8)	0.49 (0.32–0.73)	0.0005

Revascularization	12 (0.3)	5 (0.1)	0.39 (0.13–1.12)	0.08

SPC Strategy versus Control (Subgroup: SPC without Aspirin)**				

Randomized	6,020	6,041		

CV death, MI, stroke, revascularization	292 (4.9)	202 (3.3)	0.68 (0.57–0.81)	<0.0001

CV Death	149 (2.5)	110 (1.8)	0.73 (0.57–0.93)	0.01

MI	64 (1.1)	38 (0.6)	0.59 (0.39–0.88)	0.009

Stroke	91 (1.5)	57 (0.9)	0.62 (0.44–0.86)	0.005

Revascularization	70 (1.2)	39 (0.6)	0.55 (0.37–0.81)	0.003

**SECONDARY PREVENTION OF CVD**				

	**CONTROL GROUP N (%)**	**SPC GROUP N (%)**	**HR (95%CI)**	**p-VALUE**

**SPC Strategy versus Standard of Care (Composite)**				

Randomized	1,229	1,237		

CV death, MI, stroke, revascularization	156 (12.7)	118 (9.5)	0.76 (0.60–0.96)	<0.02

CV Death	71 (5.8)	48 (3.9)	0.67 (0.47–0.97)	–

MI	62 (5.0)	44 (3.6)	0.71 (0.48–1.05)	–

Stroke	27 (2.2)	19 (1.5)	0.70 (0.39–1.26)	–

Revascularization	28 (2.3)	27 (2.2)	0.96 (0.57–1.63)	–


*Table adapted from Joseph (2021) (23). The table combines data from the three randomized clinical trials that were powered to detect clinical outcomes for the SPC strategy in primary CVD prevention: HOPE-3, PolyIran, and TIPS-3, although PolyIran also included a minority of patients (~10%) with pre-existing CVD. **Patients from the HOPE-3 trial are not included in the subgroup analyses, as aspirin use was not part of this study. **Locations of enrolling sites for the primary prevention trials:** Argentina, Australia, Bangladesh, Brazil, Canada, China, Colombia, Czech Republic, Ecuador, Hungary, India, Indonesia, Iran, Israel, Malaysia, Netherlands, Philippines, Russia, Slovakia, South Africa, South Korea, Sweden, Tanzania, Tunisia, United Kingdom, Ukraine (7,8,9). **Locations of enrolling sites for the secondary prevention trials:** Czech Republic, France, Germany, Hungary, Italy, Poland, Spain (10).

An individual participant data meta-analysis of the three large RCTs (N = 18,162) confirmed that the SPC strategy reduced the composite MACE outcome of cardiovascular death, myocardial infarction, stroke, or arterial revascularization by 38% compared with usual care or placebo (23) (Table 1). The meta-analysis calculated the risk profile across all three primary prevention trials, and found participants to have intermediate to high five or 10-year predicted risk of ASCVD. It showed that the benefit of SPC treatment over placebo started within one year of follow-up, and that these SPC treatment effects were similar regardless of baseline sex, smoking status, body mass index, diabetes status, blood pressure level, or cholesterol level. The largest reductions in CVD were observed with SPCs that included aspirin, though reductions were also significant for SPCs without aspirin (Table 1). SPC treatment was well tolerated, with low rates of gastro-intestinal or intracranial bleeding that were not statistically different between intervention and control groups, regardless of aspirin use.

The benefit of SPCs on clinical outcomes was further demonstrated in a more recent study-level meta-analysis that excluded the HOPE-3 trial and included other, smaller randomized trials of SPCs that were powered for intermediate outcomes but collected MACE data. The meta-analysis found that the SPC strategy reduced all-cause mortality by 11% (N = 16,278 across four trials) and non-fatal ASCVD by 29% (N = 15,503 across five trials) compared with usual care or placebo (12).

Looking across all studies and meta-analyses, the results show that SPCs can lead to rapid, large, and consistent reductions in fatal and non-fatal MACE across a wide spectrum of people *without* prior CVD. These data support treatment initiation by clinicians and the implementation of fiscal policies for SPC reimbursement by policymakers.

In addition to benefits in primary CVD prevention, trials and resulting meta-analyses show a significant benefit of SPCs in achieving risk factor reduction in secondary CVD prevention compared with usual care (20,21,22,24). In 2022, the first large RCT (SECURE, n = 2,499) evaluated SPC in a population with cardiovascular risk factors and myocardial infarction in the prior six months. Participants were randomized 1:1 to SPC (aspirin, ramipril, atorvastatin) versus usual care with individual drugs, per local guidelines. After a median follow-up of 3.0 years, people in the SPC arm experienced a 24% reduction in the primary composite MACE outcome of CV death, nonfatal myocardial infarction, nonfatal ischemic stroke, or urgent revascularization. A similar magnitude of effect was observed for key secondary outcomes of CV death, non-fatal myocardial infarction, and nonfatal ischemic stroke (Table 1). These significant reductions in MACE were likely driven by higher adherence in the SPC group out to 24 months, compared to individual drugs (10).

The SECURE trial is another fundamental contribution to this field because it provides scientists, clinicians, patients, and policymakers with high-quality evidence to support the adoption of SPCs for secondary CVD prevention, namely people with prior myocardial infarction. However, additional studies are urgently needed to further expand the generalizability of this evidence, both to include people with other established manifestation of CVD (i.e. stroke and peripheral vascular disease, which were relatively underrepresented at <10% of the SECURE population) and to include people from LMICs (SECURE focused its recruitment in Europe) where evidence remains relatively limited (25). A recent global modeling study projected that widespread implementation of SPCs could prevent up to 130 million MACE and 72 million deaths by 2050, underscoring the substantial potential of SPC strategies to reduce cardiovascular burden, particularly in LMICs, if affordable and accessible formulations are ensured (26).

### CVD Risk Factors

Estimates of the magnitude of effect of SPCs on CVD risk factors (e.g. blood pressure or cholesterol levels) vary between RCTs. This is likely due to differences in trial characteristics, such as SPC compositions, the comparator group studied (e.g. placebo, usual care, or individual drugs), and patient characteristics. Meta-analyses of trials conducted predominantly in primary CVD prevention populations reported that SPC interventions reduced mean systolic blood pressure by 8 mmHg and low-density lipoprotein-cholesterol by 1.06 mmol/L compared with control (though these specific estimates should be viewed with caution, due to substantial heterogeneity).

In secondary CVD prevention trials, SPC interventions had smaller treatment effects on risk factors, which was likely related to greater use of individual drugs in the control groups (11,27). The reductions in intermediate endpoints with SPCs provided the expected mechanistic evidence to support the reduction in MACE that was observed in the aforementioned large RCTs. Despite these results, the understanding of the relationship between risk factor and MACE reduction remains incomplete. Further, it may not be proportional in nature, especially as the risk factor reduction observed in the large RCTs (which is likely causally associated with MACE reduction) was unexpectedly lower than the risk factor reduction observed in prior, smaller trials.

### Medication adherence

A recent meta-analysis of 11 RCTs that collectively include primary and secondary CVD prevention populations showed that drug adherence was higher with SPCs compared to individual drugs (RR = 1.16; 95% CI 1.03 to 1.29) (11). The true effect of SPCs on medication adherence is likely greater because baseline and follow-up adherence levels in the control arms of these trials were markedly higher than those observed in routine clinical practice.

Most RCTs to date have focused on adherence to SPCs for secondary CVD prevention. Limited data suggests that adherence to SPCs may be lower for primary CVD prevention compared to secondary CVD prevention; however, adherence to SPCs for primary CVD prevention is still found to be significantly higher compared to individual drugs (28,29). Improved SPC adherence for secondary CVD prevention may be expected, as prior CVD events can be powerful motivators and reminders to sustain medication, though this effect may decline over time.

## Cost-Effectiveness of SPCs

Estimating the cost-effectiveness of SPCs presents challenges due to variations in SPC formulations (and their associated costs) and variations in the costs of providing healthcare in different countries.

However, available data suggest that SPCs can be a cost-effective strategy for CVD prevention and control across countries at different income levels. A 2024 study estimated the cost-effectiveness of SPCs based on the cheapest equivalent doses using clinical outcome data from the meta-analysis of the three large RCTs in primary CVD prevention outlined above (HOPE-3, PolyIran, and TIPS-3).

The incremental cost-effectiveness ratio (ICER) for SPCs was US$ 5,767 per quality adjusted life year (QALY) in LMICs, US$ 13,967 in upper-middle income countries (UMICs), and US$ 700 in HICs. While it appears counterintuitive that the ICER ratios are lowest in the HICs, it is because the SPC drug costs are similar across countries, but the costs of the hospitalizations are much higher in HICs; therefore, the cost offsets are much greater for preventing non-fatal events. Regardless, SPCs remain cost-effective across all country income levels studied when using WHO thresholds for cost-effectiveness based on country gross domestic product per capita (15).

Similar findings were reported when estimating the cost of implementing SPC strategies in secondary CVD prevention in the United States health sector. In this model-based analysis, the ICER of an SPC consisting of aspirin, atenolol, ramipril, and atorvastatin was US$ 21,818 per QALY, making it cost-effective based on USA thresholds. Since the overall cost-effectiveness of SPCs will be highly dependent on their marketed cost, strategies should be focused on providing SPCs at costs that are similar to their cheapest equivalent components (13).

## Hurdles and proposed solutions to implementation of SPCs for CVD

Despite the strong body of evidence supporting the use of SPCs for CVD, uptake globally has been slow. Several factors within any health system can hinder widespread access to or implementation of SPCs (30). To identify the most pertinent hurdles and propose solutions, the development of this Roadmap was guided by access to medicines frameworks, published research on implementation of SPCs for CVD, insights from implementation of essential medicines for other conditions, and expert opinion (31,32). The Roadmap is structured according to the following broad categories of access to medicines – availability, affordability, and adoption (31).

Within these categories, the Roadmap focuses primarily on the public sector and identifies four key hurdles to successful implementation of SPCs:

1. Limited manufacturing of SPCs
2. Higher cost of SPCs
3. Inconsistent recommendations of SPCs in international and national guidelines and treatment protocols
4. Prescriber inertia

This section explores each of the four hurdles as they relate to availability, affordability and adoption, and proposes potential solutions to overcome them.

The hurdles identified are inter-dependent, meaning a coordinated set of strategies to address them are necessary, rather than as isolated initiatives. The four hurdles may not be the only considerations important for successful implementation of SPCs. However, these four are most likely to be relevant and specific (although not exclusive) to SPCs, rather than those that affect all health products and technologies more generally.

Other considerations relevant to all essential medicines including SPCs—for example related to supply chain forecasting, distribution, and delivery—are also important, and strategies and policies to address these should draw on documented lessons from across LMIC settings and various disease areas (33,34).

### Availability

#### Limited manufacturing of SPCs

While SPCs for hypertension are increasingly manufactured globally, SPCs for CVD continue to be manufactured exclusively in a limited number of countries by a limited number of companies. Table 2 lists all SPCs for CVDs with three or more drugs identified globally through a recent survey of SPC availability, followed by literature review and targeted expert consultations (35). While there are more two-drug SPCs available, most trials with reductions in major CVD events included three or more drugs.

**Table 2:** SPCs for CVD with three or more drugs.


COMBINATIONS	BRAND & MANUFACTURER	COUNTRY OF MANUFACTURE

**1.** Aspirin, simvastatin, ramipril, atenolol, hydrochlorothiazide	“Polycap” by Cadila	India

**2.** Atorvastatin, perindopril, amlodipine	“Triveram” by Servier	France

**3.** Aspirin, atorvastatin/simvastatin, ramipril	“CNIC Polypill”, “Trinomia”, “Sincronium”, and “Iltria” by Ferrer Internacional, S.A.	Spain

	“Ramitorva” and “Zycad” by Zydus	India

	“Polytorva” by USV	India

	“Ril AA” by East West Pharmaceuticals	India

	“Heart Pill” by Excella Pharma	India

**4.** Aspirin, atorvastatin, ramipril, metoprolol	“CV-Pill Kit” by Torrent Pharmaceutical “Zycad-4 kit” by Zydus Cadila	India India

**5.** Aspirin, simvastatin, lisinopril, atenolol	“Red Heart Pill Version 1” by Dr. Reddy’s Laboratories	India

**6.** Aspirin, losartan, atenolol, and atorvastatin	“Starpill” by Cipla	India

**7.** Rosuvastatin, candesartan, hydrochlorothiazide	“Polilep” by Lepetit	Argentina

**8.** Atorvastatin, ramipril, clopidogrel	“Atamra CV kit” by Amra Remedies	India

**9.** Aspirin, simvastatin, lisinopril, hydrochlorothiazide	“Red Heart Pill Version 2” by Dr. Reddy’s Laboratories	India

**10.** Aspirin, atorvastatin, hydrochlorothiazide, enalapril	“Polypill-E” by Alborz Darou Pharmaceutical Company	Iran

**11.** Aspirin, atorvastatin, hydrochlorothiazide, valsartan	“Polypill-V” by Alborz Darou Pharmaceutical Company	Iran

**12.** Ramipril, Atorvastatin, metoprolol	“Lifepill 3” by Zydus	India

**13.** Ramipril, Atorvastatin, Aspirin	“Eze Pill” by Welcure Pharma	India


The data were collected from a previous survey conducted by the WHF (30), and supplemented with those identified in previous literature. Additionally, WHF conducted targeted consultations, which included experts from academia and industry.

Some pharmaceutical companies that were previously heavily invested in SPC development have withdrawn from the market entirely (36). As a result, there is limited price competition, and most countries are reliant on overseas manufacturing and importation, which increase costs. In LMICs specifically, local manufacture of medications, including SPCs, may be hindered by constraints related to available expertise, technology, and infrastructure (37).

Among the reasons for the limited number of SPC manufacturers is that there are few incentives to manufacture SPCs, regardless of disease. This deficiency is attributed to “market failure”, as the current market encourages manufacturers to invest in novel, high-margin products (regardless of public health benefits) rather than in the production of established medicines (38). Although new SPCs can be patented based on the specific drug combination, manufacturing process, or delivery mechanism, their relatively low sale prices require a higher demand and unit sales to be profitable. Current levels of demand from health systems, healthcare workers, and patients appear to not be consistently high enough to encourage many brand name or generic companies to enter the SPC market for CVDs.

Manufacturers may also be deterred by complex and poorly defined pathways to regulatory approval in many countries. Like all generic medicines, regulatory approvals of SPCs require the demonstration of bioequivalence between the SPC and the corresponding individual components through pharmacokinetic and pharmacodynamic testing. This process can be technically challenging, time consuming, and costly. One challenge comes with combining multiple active pharmaceutical ingredients in a single tablet or capsule, as this may cause its biopharmaceutical and pharmacokinetic behaviour to deviate from that of the separate component products. SPCs, for example, are associated with high within-individual variability and higher likelihood for potential drug-drug interactions that may alter the absorption, distribution, metabolism, and excretion profiles of therapeutics, making it more difficult to establish bioequivalence (39). Regulators’ concerns about generalisability of data from existing RCTs to broader populations may also impede regulatory approval.

The protracted approval process manifests in the limited number of SPCs for CVDs currently approved for marketing. For example, apart from a dual combination of amlodipine and atorvastatin, no SPCs targeting multiple CVD risk factors have been approved for atherosclerotic CVD prevention in the USA to date, a situation seen in many other countries.

However, for SPCs targeting hypertension, the United States Food and Drug Administration (FDA) has somewhat eased the approval process by relaxing the mandates for clinical testing. If a sponsor provides evidence for the highest doses approved or proposed for use in combination, further studies of lower doses of the same combination are not necessary (40). Similarly, the European Medicines Agency accepts the evidence base for separate components of the SPC, if the evidence base is not generated with the SPC itself, provided that relevance and rationale can be verified (41).

### Affordability

#### Higher cost of SPCs

In most resource-limited health systems, health budgets allocated to medicine procurement are often insufficient to meet population needs, forcing governments and procurement agencies to make difficult decisions about which medications to prioritise (42). In many countries, medications must be included in national or state essential medicines lists (or formularies) and in national clinical guidelines or treatment protocols to be eligible for coverage through national health insurance or government subsidies. Where medicines are not subsidised or covered by public health insurance schemes, or are unavailable in the public sector due to lack of prioritisation or stock outs, patients without private insurance are forced to pay out-of-pocket for their medications. For many, these out-of-pocket costs are often unaffordable (43) and are a common barrier to medication adherence (44), especially in LMICs.

In some countries procurement prices for SPCs are identical to the sum of the procurement cost for individual component medications (45), or the retail prices of SPCs are at a level considered “affordable” according to WHO definitions (35). These countries include India and Spain. However, in other countries, the procurement and retail cost of SPCs is higher than the sum of individual component medications, making them unattractive to government or individual patient purchasers (46,47).

The higher prices of SPCs in many countries may be driven by the relatively low number of manufacturers, limiting cost negotiation and market competition. In some cases, the higher cost may be due to patenting. For example, while the individual components of SPCs are off-patent, manufacturers of SPCs can engage in secondary or “re-patenting”, where the specific formulation or manufacturing process is patented to enable sale at a more profitable price (48). A 2016 WHF study evaluated the patent status of 48 cardiovascular drugs globally and found that 80% of SPCs had patent protection, with most patents pertaining to novel formulations or co-formulations, or the manufacturing process (49).

In principle, determining the affordability of SPCs from a government perspective should consider the entirety of potential cost savings to the health sector attributed to improved treatment adherence and outcomes related to use of SPCs. This may include savings from avoided hospitalizations, long term chronic care, or rehabilitation services. It may also include a reduction in supply chain costs due to using one pill instead of three or four (50). As discussed above, studies that have accounted for these and other potential cost savings have found the use of SPCs to be cost-effective in the countries where they were evaluated.

### Adoption

#### Inconsistent recommendations of SPCs in international and national guidelines and treatment protocols

Global adoption of SPCs, or promotion of SPCs for CVD by internationally leading regulatory and scientific bodies, has made significant progress. The 2023 inclusion of SPCs for ASCVD prevention and control in the WHO Model List of Essential Medicines is a step toward promoting their adoption in leading international guidelines (51). Indeed, as a strategy to improve adherence and treatment outcomes, SPCs are now included for secondary prevention among “hypertensive dyslipidemic patients at elevated CV risk” in the 2023 European Society of Hypertension guidelines and in the 2023 European Society of Cardiology guidelines for the management of acute coronary syndromes (52,53). However, several leading international guidelines still do not include SPCs for management of CVD.

Inclusion of a product in the WHO Model List of Essential Medicine can stimulate inclusion in national EMLs and formularies, though this is often a slow process. Cross-country comparisons, for example, have reported substantial gaps in the selection of medicines at the national level for inclusion in EMLs compared with those recommended by the WHO (54). A WHF survey conducted in 2022 reported that SPCs for CVD were not listed in the national EMLs of any of the countries that manufactured them (where EMLs exist) (35). Delays in the inclusion of SPCs in national lists and formularies result in their delayed adoption in national clinical guidelines, programs, and procurement lists for public sector facilities, especially in primary and secondary care. This may exacerbate existing health inequities, as people with lower socio-economic backgrounds are often more dependent on public sector medication provision or subsidisation.

Delays to adopt SPCs into national lists may be in part due to a reluctance to include medications that are not affordable to purchasers or governments, or likely to be readily available, underscoring the cyclical and interdependent nature of the hurdles outlined in this Roadmap.

#### Prescriber inertia

Adoption of SPCs among prescribers globally has been slow (55,56). Poor adoption may be down to several factors. First, as discussed above, SPCs are not consistently included in national guidelines, which in many contexts dictate treatment. Second, providers are often unwilling to prescribe outside of what they know is available or with which they are familiar (57,58). Third, poor adoption of SPCs among providers may be driven by concerns about potential side-effects and dosing inflexibility (55). In primary prevention specifically, SPCs have been associated with “overmedicalization” (59). Although the age-only screening and mass treatment approach proposed by Wald and Law in 2003 when they introduced the concept of the “polypill” has not been widely accepted, the perception still exists that SPCs for primary prevention among healthy populations may undermine non-medical interventions and unnecessarily expose patients to risks of adverse events (16).

A 2024 cross-sectional survey report focused on anti-hypertensive SPCs—involving 179 prescribers across 24 countries—suggested that cost to the medical practice was the leading barrier to prescription of SPCs. This was followed by prescribers’ lack of trust in clinic-measured blood pressure, and inadequate access to SPCs among their patients. The survey found that prescriber educational support, including feedback on prescribing patterns among peers, could be the major facilitator to enhance SPC prescribing. These findings reaffirm that addressing prescriber inertia requires both interventions directed towards prescriber education and awareness, and system-level reforms (60).

### Proposed solutions

Table 3 outlines potential solutions for each of the four hurdles discussed above and identifies further barriers not discussed in detail in this Roadmap. No single solution will be sufficient to improve implementation of SPCs and any effort must involve cooperation among multiple stakeholders at all levels of the health system.

**Table 3:** Roadblocks and Potential Solutions to SPC Implementation for CVDs.


ACCESS CHALLENGES	ROADBLOCKS	PROPOSED SOLUTIONS

**AVAILABILITY**	**Limited manufacturing**	Financial incentives to attract manufacturers to the market e.g. subsidies, tax breaks, advanced purchase agreements Training and technology transfer programs from current manufacturers to new manufacturers, especially those in LMICs Investment in local manufacturing infrastructure through grants, loans, and partnerships Early engagement of manufacturers with regulatory agencies and technical assistance to manufacturers to navigate regulatory processes Harmonisation of guidelines across different health regulatory agencies

	*Others:* *Lack of data on need, supply, demand, and price-elasticity; supply chain weaknesses (excessive fragmentation, poor visibility of stock, erratic funding flows, poor governance)*	Strengthen data systems and streamline supply chains, through sustainable financing mechanisms

**AFFORDABILITY**	**Higher cost of SPCs**	Large scale-pooled procurement at the national or regional level to improve purchasing power and reduce costs*

	*Others:* *Gaps in health insurance scheme coverage of people with NCDs*	Inclusion of SPCs in national insurance coverage or reimbursement lists

**ADOPTION**	**Inconsistent recommendations of SPCs in international and national guidelines and treatment protocols** **Prescriber inertia**	Development of context-specific effectiveness and cost-effectiveness evidence Multi-stakeholder collaborations (e.g. researchers, advocacy organisations, policy makers, professional societies) to support the translation of clinical trial and real-world research evidence into clear policy that is consistent across policy institutions (62). For example, inclusion of SPCs on essential medicines lists and as recommended first line treatment in guidelines/treatment protocols Ongoing capacity strengthening and mentoring initiatives to support healthcare workers to integrate SPCs into existing practices Education and awareness campaigns by professional societies and Ministries of Health aimed at healthcare providers, using evidence (including feedback on prescribing patterns among peers) to show flexibility and benefits of SPCs to healthcare workers and patients

	*Others:* *Clinical or institutional inertia or barriers and lack of awareness of SPCs among patients*	Use of implementation research tools to co-design context-specific implementation strategies in each country Inclusion of all relevant stakeholders (including health workers and pharmacists) in co-production and implementation research studies


*The recent HEARTS in the Americas initiative of the Pan-American Health Organization may offer some insights for pooled procurement of CVD medications. The initiative included centralised pooled procurement of SPCs for hypertension among several countries in Latin America through a Strategic Fund, aiming to consolidate regional demand and achieve competitively priced long term agreements for procurement of quality generic products (61).

## Conclusion

This Roadmap highlights the well-established safety and efficacy of SPC therapy for primary and secondary prevention of CVDs. Despite the benefits of SPCs, global adoption and implementation remain limited, particularly in LMICs where the need is greatest. The Roadmap proposes several solutions to address some of the major challenges identified—e.g. limited manufacturing capacity, higher costs of SPCs, inconsistent guideline recommendations, and prescriber inertia. Solutions include financial incentives and technology transfer programs for manufacturers, integration of SPCs into national insurance and reimbursement schemes, stronger advocacy by international and national cardiac societies for inclusion of SPCs in national guidelines, and targeted education for healthcare providers and patients. Given the variability in healthcare systems and economic conditions, implementation strategies should be adapted to regional and national contexts to ensure maximum impact. Overcoming these barriers is critical to expanding access to SPC therapy and supporting efforts to reduce the global mortality and morbidity burden of CVDs.
